# A cross-sectional evaluation of the Dutch RHAPSODY program: online information and support for caregivers of persons with young-onset dementia

**DOI:** 10.1016/j.invent.2022.100530

**Published:** 2022-03-26

**Authors:** Maud Daemen, Jeroen Bruinsma, Christian Bakker, Rob Groot Zwaaftink, Raymond Koopmans, Andrea Oostijen, Bernard Loose, Frans Verhey, Marjolein de Vugt, Kirsten Peetoom

**Affiliations:** a Department of Psychiatry and Neuropsychology/Alzheimer Center Limburg, School for Mental Health and Neuroscience, Maastricht University, Maastricht, the Netherlands; b Department of Primary and Community Care, Radboud University Medical Center, Nijmegen, the Netherlands; c Radboudumc Alzheimer Center, Nijmegen, the Netherlands; d Groenhuysen, Center for Specialized Geriatric Care, Roosendaal, the Netherlands; e Dutch Alzheimer's Society, Amersfoort, the Netherlands; f Joachim and Anna, Center for Specialized Geriatric Care, Nijmegen, the Netherlands

**Keywords:** Young-onset dementia, Caregiver, Web-based, E-learning, Education

## Abstract

Caregivers of persons with young-onset dementia (YOD) have an explicit need for tailored information and support about YOD. Therefore, during the European RHAPSODY project a web-based information and support program for YOD caregivers was developed. The program was recently tailored to the Dutch context. This study evaluates the Dutch version on user acceptability, usability, user satisfaction, and user behavior.

**Methods:**

A cross-sectional study was conducted to evaluate the publicly available Dutch RHAPSODY program. A pop-up survey, extensive survey, and a semi-structured interview were used to evaluate how visitors perceived the program in terms of acceptability, usability, and their satisfaction. Web metrics registered user behavior. Quantitative data were analyzed using descriptive statistics and a deductive content analysis was used to analyze qualitative data.

**Results:**

A total of 26 participants completed the pop-up survey, 19 completed the extensive survey, and 10 participated in the semi-structured interviews. Most participants were caregivers and healthcare professionals. They perceived the program as acceptable and usable in daily life and were satisfied with the quality of the content. The majority would use the program again and recommend it to others. Participants emphasized the necessity and desirability of a central platform incorporating educational and practical information about YOD. The page with an explanation about what YOD entails was most viewed (360 unique page views). Most time was spent on the page about the diagnostic process (6.5 min).

**Conclusions:**

The Dutch RHAPSODY program showed good user acceptability, usability, and user satisfaction. The program met the need for tailored information and support regarding YOD and adds value to existing available support for YOD caregivers. Raising awareness about the program's existence among healthcare professionals may help caregivers to find appropriate post-diagnostic information. The program also provides educational opportunities for healthcare professionals.

## Highlights

• The RHAPSODY program is tailored to the Dutch context, and is publicly available.
• The program met the informational needs of young-onset dementia caregivers.
• Participants reported the program is acceptable and usable in daily life.
• The program adds value to existing available support for caregivers.
• Involving stakeholders in an early phase contributes to sustainable implementation.

## 1 Introduction

The first symptoms of young-onset dementia (YOD) have an onset before the age of 65 years (Koopmans and Rosness, 2014; van de Veen et al., 2021). Two of the most common types of YOD are Alzheimer's dementia and frontotemporal dementia (Hendriks et al., 2021). Unlike dementia symptoms in late-onset dementia (LOD), the presentation of symptoms in YOD is more varied due to the larger variety in underlying pathology and differences in clinical manifestation (Rossor et al., 2010). For example, behavioral symptoms occur more often in YOD, including changes in social abilities, apathy, impulsivity, or uninhibited behavior (Ducharme and Dickerson, 2015; Kuruppu and Matthews, 2013). Partly, this is due to the higher prevalence of frontotemporal dementia in YOD, compared to LOD (Onyike and Diehl-Schmid, 2013; Rosness et al., 2016). Additionally, Alzheimer's dementia in individuals younger than 65 years is more likely to be characterized by non-memory symptoms compared to dementia at an older age, such as language deficits and executive dysfunctions (Barnes et al., 2015; Koedam et al., 2010).

Coping with these symptoms poses unique challenges for caregivers of persons with YOD and can result in high levels of burden, distress, and depressive symptoms (Ducharme et al., 2013; Lim et al., 2018; Millenaar et al., 2014). Spousal caregivers are often in their 50s, they may be employed, and can have children living at home (Cartwright et al., 2021; Grundberg et al., 2021). This can result in difficulty in balancing the caregiving role with professional responsibilities, or financial problems due to reduced working hours or early retirement (Ducharme et al., 2013; Hvidsten et al., 2019; Mayrhofer et al., 2021; van Vliet et al., 2010). Additionally, caring for a family member with YOD is known to result in profound shifts in family roles and relationships (Cabote et al., 2015; Svanberg et al., 2011).

Caregivers experience significant difficulty in coping with these circumstances and express an explicit need for age-appropriate professional support (Arai et al., 2007; Bannon et al., 2021; Lockeridge and Simpson, 2013; Millenaar et al., 2014; Millenaar et al., 2016). Currently, YOD caregivers often experience most information is too generic and focused on caregivers of persons with dementia in old age (Bannon et al., 2021; Cations et al., 2017; Mayrhofer et al., 2018; Millenaar et al., 2016). Especially following the diagnosis, caregivers have a need for specific information about what YOD entails, the prognosis, advice on informing others, questions regarding heredity, and available support and care. They also prefer to have practical information, for example on coping with symptoms and obtaining support for financial problems (Ducharme et al., 2014; Mayrhofer et al., 2018; Millenaar et al., 2016; Rosness et al., 2012). Psychoeducation is an opportunity to adequately inform caregivers and may help them to acquire coping skills (Cations et al., 2017; Spreadbury and Kipps, 2018). Potentially, psychoeducation could also be used to inform healthcare professionals. This is important since previous research has shown that they, in general, tend to have limited knowledge about YOD (Bruinsma et al., 2020; Spreadbury and Kipps, 2018).

Studies so far have demonstrated that web-based services are flexible, easily accessible, and cost-effective, especially in areas hindered by geographical barriers (Godwin et al., 2013; Klimova et al., 2019; Waller et al., 2017). Previously, the European RHAPSODY consortium developed a web-based information and support program for YOD caregivers (Kurz et al., 2016). A pilot study in Germany, France, and the United Kingdom showed positive results regarding user acceptability, program satisfaction, and caregiver well-being (Metcalfe et al., 2019). Building upon these promising results, we tailored the program's content for use in the Dutch context. The current study evaluates the Dutch RHAPSODY program in terms of user acceptability, usability, user satisfaction, and user behavior.

## 2 Methods

This cross-sectional study included quantitative and qualitative data collection methods, to evaluate user acceptability, usability, user satisfaction, and user behavior of the Dutch RHAPSODY program. Between October and December 2020, online surveys and semi-structured telephone interviews were conducted. Additionally, web metrics such as the number and duration of page visits were registered. The online program was freely and publicly available via the website of the Dutch Alzheimer's Society [*Alzheimer Nederland*], www.dementie.nl [*Online training dementie op jonge leetijd*]. The guidelines of the CONSORT-EHEALTH were used for reporting the results of this study (Eysenbach, 2011).

### 2.1 Development of the Dutch RHAPSODY program

The European RHAPSODY consortium consists of partners from six countries. The Dutch RHAPSODY program was built upon the previously developed versions designed and evaluated for caregivers of persons with YOD (Kurz et al., 2016; Metcalfe et al., 2019). As in the previous versions, YOD caregivers are the primary target group of the Dutch program. The program provides separate chapters describing different topics, such as the medical background of YOD, management of challenging behavior, dealing with changes in role patterns within the family, access and availability of appropriate support, and (re)gaining a sense of balance during the caregiving trajectory. In line with the previous versions, the Dutch program (see Appendix A) contains similar textual information, explanatory pictures, animations, and reflective questions. The research team translated, revised, and tailored the content of the previous RHAPSODY versions to the Dutch context. New video vignettes were developed featuring three family caregivers and two YOD experts (CB and MdV). Also, one chapter was completely rewritten to provide information on dedicated post-diagnostic care and support for YOD available in the Netherlands. Caregivers, healthcare professionals, and field experts provided feedback and input during this tailoring process. Additionally, specific attention was devoted to the use of understandable language for the general public and lay audience.

### 2.2 Recruitment

**Fig. 1:**
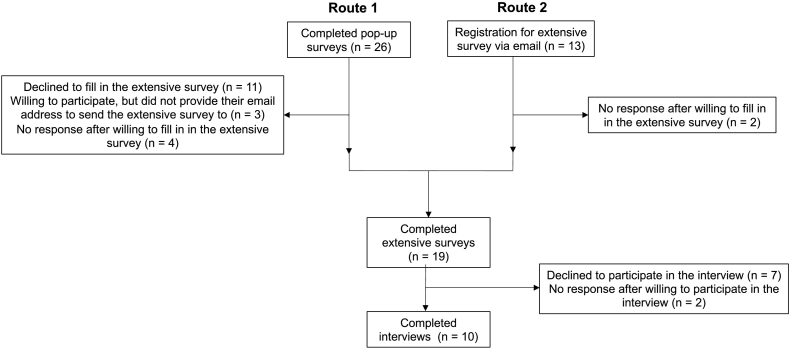
Routes for participation flow diagram.

Both YOD caregivers and healthcare professionals involved in YOD care were invited between October and December 2020 to use the online program and to participate in our study to evaluate user acceptability, usability, user satisfaction, and user behavior. Because the development of the Dutch RHAPSODY program was based on the previous RHAPSODY programs and its corresponding pilot study (20 participants in each country), the aim was to recruit 15–20 participants (Metcalfe et al., 2019). Information about the program was spread via newsletters, social media, presentations, flyers, and by using personal mailing lists of Alzheimer Center Limburg, Radboudumc Alzheimer Center, the Dutch Alzheimer's Society, the Dutch YOD Knowledge Center [*Kenniscentrum Dementie op Jonge Leeftijd*], and the Academic Network of Nursing Homes Nijmegen [*UKON*]. All visitors who accessed the program were automatically invited to complete a short pop-up survey that appeared if they visited a page longer than 30 s (Fig. 1, see route 1). The main purpose of the pop-up survey was to invite visitors to complete an additional and more extensive survey. Subsequently, if they were willing to participate in the extensive survey, their email-address was requested in order to send them the link to this survey. Participants were also able to contact the research team directly to request participation (Fig. 1, see route 2). All participants who completed the extensive survey were invited for an in-depth interview that was conducted via telephone.

### 2.3 Measurements

#### 2.3.1 Participant characteristics

Participants were asked about their background in the pop-up survey. In the additional, extensive survey, more detailed questions were asked about either their relationship to the person with YOD or about their healthcare profession. Participants were also asked about their gender and age.

#### 2.3.2 Exploring user acceptability, usability, and user satisfaction

Quantitative and qualitative measures provided insight in user acceptability, usability, and user satisfaction. User acceptability was defined as the perceived ease of use and usefulness of the Dutch RHAPSODY program (Rahimi et al., 2018). Similar to the study evaluating previous versions of the RHAPSODY program, four subscales of the Technology Acceptance Model (TAM) were used (Metcalfe et al., 2019) to measure user acceptability. The TAM predicts individual adoption of internet driven technology, and can explain around 40% of the variance in the actual use and intention to use (Venkatesh and Bala, 2008). The TAM examines technology in terms of perceived usefulness, ease of use, behavioral intention to use, and computer self-efficacy. The current study assessed these four domains in the extensive survey using a 7-point Likert scale, ranging from 1 ‘totally disagree’ to 7 ‘totally agree’.

Usability was defined as the extent to which the Dutch RHAPSODY program could be used in daily life and meet the needs of the target group (Dix et al., 2003). To illustrate, usability was examined in the extensive survey by asking if participants found it difficult to allocate time to embed the program in daily life. Additionally, questions focused on the quantity of information provided. Usability items were rated on a 5-point Likert scale, ranging from 1 ‘totally disagree’ to 5 ‘totally agree’.

User satisfaction referred to the quality and relevance of the program's content and likelihood of recommending the Dutch RHAPSODY program to others. Similar to the study performed by Metcalfe et al. (2019), user satisfaction was assessed by asking about the perceived quality, relevancy, understandability, applicability of content in daily life, and layout of the program. Items in the extensive survey were rated on a 5-point Likert scale, ranging from 1 ‘very bad’ to 5 ‘very good’. One item in the pop-up survey measured if the program helped in coping with dementia, and was rated on a 5-point Likert scale ranging from 1 ‘totally disagree’ to 5 ‘totally agree’. Another item in the pop-up survey measured the likelihood of recommending the program to others, rated on a scale from 1 ‘very unlikely’ to 10 ‘very likely’.

The in-depth and semi-structured interviews were audio-recorded and conducted via telephone by two researchers (MD and KP). Interviews lasted around 40 min. A semi-structured interview guide was used to provide insight in user acceptability, usability, and user satisfaction (see Appendix B). For example, participants were asked how they perceived the quality of the content, language use, usefulness of topics, the likelihood of that they would recommend the program to others, and how they experienced navigating through the program content.

#### 2.3.3 User behavior

Web metrics were collected anonymously to investigate the behavior of visitors in terms of the number and duration of page visits. Due to settings of the web page, a visitor would count as a unique page visitor once every 24 h. Meaning that if a visitor returns to the program after 24 h, a new unique page view was registered. Web metrics were also gathered three months after the evaluation study to monitor the implementation of the Dutch RHAPSODY program.

### 2.4 Data analysis

Quantitative data was analyzed using SPSS Statistics version 27. Descriptive statistics were calculated (mean, mode, and standard deviation) to explore participant characteristics, user acceptability, usability, user satisfaction, and to analyze the web metrics. The qualitative data was analyzed using a deductive content analysis, using interview transcripts (Elo and Kyngäs, 2008). Qualitative data was examined from a pragmatic theoretical perspective, examining whether the program met the needs of YOD caregivers (Kaushik and Walsh, 2019). Therefore, transcripts were deductively coded by the first author (MD) using Atlas.Ti version 9.0.7. Based on the previous pilot study (Metcalfe et al., 2019), relevant themes (user acceptability, usability, and user satisfaction) were identified and guided the coding process. Subsequently, themes and associated codes were summarized in a thematic mind-map and discussed with KP to verify the results.

### 2.5 Ethical considerations

Pop-up surveys are structurally used by the website of the Dutch Alzheimer's Society to monitor user satisfaction, no consent form was needed. Prior to participation in the extensive survey and in-depth interview, participants received an information letter by email and gave online informed consent for participation. The study protocol was approved by The Faculty of Health, Medicine and Life Sciences Research Ethics Committee of Maastricht University (FHML-REC/2020/090).

## 3 Results

A total of 26 participants completed the pop-up survey. Next, 19 participants completed the extensive survey, and 10 participated in the in-depth interviews. The two recruitment routes are shown in Fig. 1.

### 3.1 Participant characteristics

The majority of participants who completed the pop-up survey had a relative with dementia (53.8%) or were professionally involved with dementia (46.2%). A varied sample of participants completed the extensive survey and participated in the in-depth interviews, such as spouses, children, or other relatives of persons with different YOD-subtypes, and healthcare professionals with different backgrounds (Table 1).

**Table 1:** Participant characteristics.

		Extensive survey (*n* = 19)	Interview (*n* = 10)
Caregivers		N (=10)	N (=7)
Gender	Male	5	4
	Female	5	3
Age	30–39 years	2	1
	40–49 years	0	0
	50–59 years	4	3
	60–65 years	2	1
	>65 years	2	2
Relationship to person with dementia	Spouse	6	4
	Brother/sister	2	2
	Father/mother (in-law)	1	1
	Not specified	1	0
Diagnosis of the person with dementia	Alzheimer's dementia	6	4
	Frontotemporal dementia	2	2
	Not specified	2	1
Years since diagnosis	<3 years	2	1
	3–5 years	4	4
	>5 years	2	1
	Not specified	2	1
Healthcare professionals		N (=6)	N (=2)
Gender	Male	0	0
	Female	5	2
	Not specified	1	0
Age	40–49 years	1	0
	50–59 years	2	1
	60–65 years	2	1
	> 65 years	1	0
Background	Case manager	2	0
	Dementia care coordinator	1	1
	Dementia counselor	2	1
	Volunteer	1	0
Other		N (=3)	N (=1)
Gender	Male	0	0
	Female	3	1
Age	40–49 years	1	0
	50–59 years	2	1
Background	Both caregiver and healthcare professional	1	0
	Person with Alzheimer's dementia	1	1
	Not specified	1	0

### 3.2 User acceptability

Most participants who completed the extensive survey had high levels of computer self-efficacy (Table 2). The majority strongly agreed the program was useful, easy to use, and indicated they would use it again.

**Table 2:** Scores on user acceptability.

Technology acceptance model	Mean (SD)	Range
Perceived usefulness	6.42 (1.39)	1–7
Ease of use	5.95 (1.13)	1–7
Behavioral intention to use	5.63 (1.30)	1–7
Computer self-efficacy	6.11 (0.81)	1–7

In the in-depth interviews, participants expressed they had mixed feelings regarding the ease of use. Some experienced the navigation within the program as clear, whereas others experienced it as confusing. Participants suggested minor improvements to enhance the navigation. For example, a clear home-button redirecting to the landing page, a table of contents at the start of each chapter, and providing clarification if a hyperlink led to an external web page.

‘Sometimes it was hard to know where you were in the program. I wondered how to go back to the landing page, especially if a link led me to an external web page.’

- Dementia care coordinator

### 3.3 User usability

Nine out of 19 participants (47.4%) who completed the extensive survey agreed it was challenging to find time to follow the program, five participants (26.3%) disagreed, and the remaining five (26.3%) neither agreed nor disagreed. Furthermore, two-thirds of the participants reported the level of detail, number of chapters, duration of chapters, and overall length of the program as exactly right, while the remaining one-third rated it as a lot.

Participants valued the possibility to follow the program at any time and any location. Although most participants indicated the program contained comprehensive information, it was highlighted that this is necessary for a topic like YOD. According to participants, the wide range of information provided a reason to return to the program at a later stage when other topics are applicable.

‘The program contains comprehensive information, but all the information is needed. YOD is a complex and diverse disease.’

- Dementia counselor

Furthermore, participants valued the language use in the program, and expressed it was understandable for those with and without a medical background. They felt the Dutch RHAPSODY program was relevant for YOD caregivers and healthcare professionals but also for persons not directly involved with YOD. For example, to create awareness and understanding about YOD among employers.

### 3.4 User satisfaction

Participants who completed the extensive survey were highly satisfied with the overall quality of the program, as 94.7% rated the quality as good to excellent. Participants valued the relevance, understandability, and applicability of the content in daily life. Moreover, they rated each individual chapter and the reflective questions as useful. Approximately 80% of participants rated the overall layout and the layout of each individual chapter as clear. On the pop-up survey, 21 of 26 participants (80.8%) agreed or totally agreed the program helped in coping with YOD in daily life. The vast majority would recommend the program to others (Mean = 8.0, SD = 1.3, on a scale from 1 to 10). Qualitatively, participants highlighted the need for a program including educational and practical information about YOD bundled in one place.

‘As a caregiver, there is always the question ‘where do I find all the information?’. You can't see the forest for the trees. After looking into this program, this is the place where I would recommend people visit.’

- Daughter of a person with Alzheimer's dementia

Participants experienced the alternation between texts and videos as pleasant. The great usefulness and applicability of practical tips were mentioned serval times during the interviews. Furthermore, participants recognized the information in their own daily experience and felt it retrospectively enhanced their understanding of symptoms of YOD. Participants were satisfied with the variety of topics covered in the program, especially regarding different subtypes of YOD, coping with behavioral symptoms, and availability of care and support services. Nevertheless, several participants felt they had already obtained some of the information themselves in the past years. Thus, not all information was still relevant anymore.

‘At the beginning we searched for a lot of information. If only I had this training before, it would have been of great help, also to better understand my wife's behavior.’

- Husband of a person with frontotemporal dementia

‘The tips provided in the program are good. Not only for caregivers but also for persons with dementia themselves.’

- Person with Alzheimer's dementia

### 3.5 User behavior

The landing page of the Dutch RHAPSODY program registered 2461 unique page views between October and December 2020 (see Appendix C). The page with an explanation about what YOD entails was most viewed and registered 360 unique page views. Visitors had spent most time reviewing content about the diagnostic process (6.5 min). The number of unique page views gradually decreased from the first to the last chapter. Overall, this same pattern was seen within the different pages of each individual chapter. In the three months after evaluating the program, the landing page registered 1855 unique page views.

## 4 Discussion

Our quantitative and qualitative findings demonstrate a good user acceptability, usability, and user satisfaction of the Dutch RHAPSODY program. Participants indicated they would use the program again and would recommend it to others. In-depth interviews emphasized the high need for education and tailored information about YOD to be collected in one place, which the RHAPSODY program provided. Both caregivers and healthcare professionals were satisfied with the quality of the content and topics, such as information on different YOD-subtypes, coping with behavioral symptoms, and available care and support services. Minor suggestions for improvement were identified, such as enhancing the navigation within the program.

Participants valued the usefulness, relevance, and applicability of the content in daily life. These results are consistent with the previous study on other versions of the RHAPSODY program (Metcalfe et al., 2019). For YOD caregivers, flexibility is an important aspect of using support programs (Bannon et al., 2021; Cations et al., 2017). This also applied to the RHAPSODY program which allowed participants to visit it from a place and time of their convenience. The findings of the qualitative interviews suggest that the Dutch RHAPSODY program adds value to already existing support, as the program met the information needs of YOD caregivers and healthcare professionals.

Approximately one-third of the participants reported that the program contained a considerable amount of information, which may explain why nine out of the nineteen persons agreed that it was difficult to allocate time to follow it. For caregivers, it can be difficult to find time to follow such a program as they already have to balance caregiving with other responsibilities, such as work, raising children, and maintaining social relationships (Ducharme et al., 2013; Hvidsten et al., 2019). Interviewees revealed that a table of contents would facilitate more direct access to the desired information and would improve the navigation through the program content. Furthermore, participants highlighted that it was pleasing to receive information tailored for different YOD-subtypes. Previous research has confirmed that caregivers have a need for YOD-subtype specific information (Bannon et al., 2021; Bruinsma et al., 2021b; Rosness et al., 2008). As known, support mainly focuses on Alzheimer's dementia or older peers (Cations et al., 2017; Mayrhofer et al., 2018). In line with previous research, web metrics showed that the page with an explanation about what YOD entails was most viewed. Caregivers, including children, desire such information to obtain a better understanding of the disease (Millenaar et al., 2014). In addition, most time was spent on the page about the diagnostic process. Diagnosing YOD is often highly complex and is associated with uncertainty for caregivers (van Vliet et al., 2011).

The Dutch RHAPSODY program was launched and evaluated during the COVID-19 pandemic, a time when there was a heightened need for and usage of digital support programs for caregivers of persons with dementia (Cuffaro et al., 2020; Dourado et al., 2020). The pandemic may therefore have facilitated familiarization with digital services (Cuffaro et al., 2020). Due to the program's free and public availability through the Internet, the program could be visited regardless of restrictions imposed by the COVID-19 pandemic. The program was co-designed and hosted by the Dutch Alzheimer's Society via its website (www.dementie.nl). This partnership and adapting the program to the wider implementation context contributes to its sustainable implementation, which facilitates translating interventions into practice (Christie et al., 2018). Our web metrics confirmed that the program continued to have visitors after finishing the evaluation study.

In order to allow timely access to age-appropriate information regarding YOD, it is recommended that caregivers be made aware of the program after their relative has received the diagnosis. Our findings reveal that participating caregivers had spent considerable time searching for reliable information online beforehand. Therefore, creating greater awareness about the Dutch RHAPSODY program is an important future direction. Healthcare professionals involved in the early phase of the caregiver trajectory, such as dementia case managers, play an essential role in getting the right information at the right time to the right caregiver. Their actively and personally informing of caregivers about the program will likely stimulate caregivers to use it. This may also improve adherence, which is often limited in web-based programs (Kelders et al., 2012). Other dissemination activities include spreading information leaflets, embedding the program at a prominent spot at the website of the Dutch Alzheimer's Society, and making referrals to the program from other (online) YOD information sources.

Although web-based programs are considered as easily accessible, previous research indicates that online support should be offered in addition, and cannot entirely replace face-to-face support of a healthcare professional (Hopwood et al., 2018; Huis in het Veld et al., 2018). The RHAPSODY program could serve as a stepping stone for caregivers to participate in other support options. For example, in the Netherlands, tailored support is available that blends a web-based approach with personal coaching, namely the Partner in Balance intervention (Bruinsma et al., 2021a; Bruinsma et al., 2021b). Lastly, although the current study showed good user acceptability, usability, and user satisfaction of the Dutch RHAPSODY program, a direction for future research would be to explore the long-term effects on caregiver well-being and coping skills.

Our findings show that nearly half of the participants who completed the pop-up survey were healthcare professionals. This percentage is relatively high considering that caregivers are the primary target group of the program. This may indicate there is an educational need among healthcare professionals regarding YOD as well. Previous studies demonstrated that educational e-learning programs allow healthcare professionals to boost their knowledge (Delf, 2013). Therefore, e-learning plays an important role in (inter)professional education on YOD (Casimiro et al., 2009; Menard and Varpio, 2014). Involving care organizations affiliated with the YOD Knowledge Center in the Netherlands could facilitate the use of the Dutch RHAPSODY program for the education of healthcare professionals. Ultimately, this would improve the quality of care and support they provide to YOD caregivers. Similarly, embedding the Dutch RHAPSODY program in (inter)professional education programs on YOD offered by health academies and universities of applied science in the Netherlands would also enable healthcare professionals to become acquainted with the program.

### 4.1 Strengths and limitations

An important strength of the Dutch RHAPSODY program is that it was based on an international evidence-based program. This allowed us to cost-effectively dedicate our resources to tailor it to the Dutch context in close collaboration with caregivers, healthcare professionals, and field experts. Another strength is that the current study allowed all visitors who accessed the program to participate in the evaluation study. No strict inclusion or exclusion criteria were defined as the program was freely and publicly accessible. This allowed for various ways of recruiting, for example via newsletters, social media, and flyers. For sustainable implementation, the program was embedded on the well-visited website of the Dutch Alzheimer's Society. Communication about this helped to raise awareness about the program's existence in a large audience of caregivers and healthcare professionals. Additionally, it ensures free, public, and structural access to the program.

The current study is accompanied by some limitations, such as the relatively small and heterogeneous sample, that may impede the generalizability of the results. Partly, the limited number of responses may be the result of visitors clicking away the pop-up survey. Participants who clicked away the pop-up survey did not receive a reminder. Moreover, the pop-up survey only appeared if participants visited a page longer than 30 s. In addition, although the web metrics are a valuable contribution to the study, they should be interpreted with caution. For example, the duration of page visits is difficult to interpret as the amount and type of information per page differed, there is no insight into whether visitors viewed the program on different devices, or if they paused the online program while using it.

## 5 Conclusions

The Dutch RHAPSODY program provides an opportunity to educate and inform YOD caregivers sustainably. Findings indicated good user acceptability, usability, and user satisfaction. The program met the information needs of YOD caregivers, and the provided information was useful in daily life. Improvements are made by facilitating better navigation within the program, including providing a table of contents and a clear home-button redirecting to the landing page. Furthermore, the RHAPSODY program has potential as an educational tool for healthcare professionals. Therefore, raising awareness about the RHAPSODY program among early stage YOD caregivers and their healthcare professionals is recommended. The partnership with the Dutch Alzheimer's Society is important as it facilitates the sustainable implementation of the program. A future research recommendation would be to reuse existing interventions that have shown positive effects in supporting YOD caregivers, and tailor them to its wider implementation context. In order to ensure that there is a comprehensive and varied range of support tailored to the specific needs of the caregivers.

## Funding

The project was funded by The 10.13039/501100001826Dutch national Organisation for Health Research and Development [ZonMw] and the Dutch Alzheimer's Society [Alzheimer Nederland]. RHAPSODY is a EU Joint Program Neurodegenerative Disease Research project.

## Declaration of competing interest

The authors declare that they have no known competing financial interests or personal relationships that could have appeared to influence the work reported in this paper.

## Appendix A Supplementary data

Appendix A Six chapters incorporated in the Dutch RHAPSODY program. Appendix A

Appendix B Semi-structured interview guide. Appendix B

Appendix C User behavior between October and December 2020. Appendix C
