# Supplementary material for: A cross-sectional evaluation of the Dutch RHAPSODY program: online information and support for caregivers of persons with young-onset dementia
Source: Internet Interv. 2022 Mar 26;28:100530. doi: 10.1016/j.invent.2022.100530 (PMC9005959; doi:10.1016/j.invent.2022.100530)
Supplement: Appendix A — Six chapters incorporated in the Dutch RHAPSODY program. [file mmc1.docx]

**Appendix A.**

**Table A.1**: Six chapters incorporated in the Dutch RHAPSODY program

| Chapter | Topics |
| --- | --- |
| **Introduction about YOD**  *General information about YOD.* | - What is YOD? How does it differ from late-onset dementia? - Medical background about the different brain functions and consequences of impairment - Diagnostic process - Heredity of YOD - Most common subtypes of YOD: Alzheimer’s dementia, frontotemporal dementia, Lewy body dementia, and vascular dementia - Medical and non-medical treatment options |
| **Problems and solutions**  *Tips on coping with daily challenges.* | - Cognitive symptoms such as memory deficits and changes in social behavior - Coping with difficulties in executing daily activities - Physical health and bodily functions |
| **Behavioral changes**  *What are behavioral changes? Tips on coping.* | - Behavioral changes and causes - Recognizing emotional symptoms - Changes in social behavior - Lack of initiative and passive behavior - Physical behavior including repetitive behavior, agitation, and aggressive behavior - Perceptions on reality and hallucinations |
| **Impact on family members**  *Changing family roles and suggestions to improve communication.* | - Changes in family roles and responsibilities - Consequences for children and suggestions on supporting them - Talking about YOD and accompanying changes with family members - Coping with emotions such as guilt, anger, and grief |
| **Self-care**  *Self-reflection, and balance caregiving with personal life.* | - Experienced burden and preventing distress - Finding a balance between caregiving and personal needs - Having unrealistic expectations towards yourself - Focus on activities that are still possible to undertake - Searching and accepting support |
| **Available support in the Netherlands**  *Where to find appropriate care?* | - Specialized YOD healthcare and availability in the Netherlands - Explanation about the roles of healthcare professionals involved after obtaining the YOD diagnosis - Available types of care and support for persons with YOD - Available types of care and support for caregivers - An overview of the relevant laws and legislation - Care arrangements, including daycare, advanced care planning, and financial matters - Arranging nursing home admission - Arranging practical issues after passing away |
|  | |
