# Supplementary material for: A cross-sectional evaluation of the Dutch RHAPSODY program: online information and support for caregivers of persons with young-onset dementia
Source: Internet Interv. 2022 Mar 26;28:100530. doi: 10.1016/j.invent.2022.100530 (PMC9005959; doi:10.1016/j.invent.2022.100530)
Supplement: Appendix B — Semi-structured interview guide. [file mmc2.docx]

**Appendix B.**

**Table B.1**: Semi-structured interview guide

| **Main questions** | **Subtopics** |
| --- | --- |
| How did you experience the use of the online program? |  |
| What do you think about the content of the online program? | - Which chapters did you follow? - Was the information relevant for your situation? - What do you think of the tips provided in the program? - What do you think of the amount of information provided? |
| What do you think of the understandability of the program? | - Which parts were easy or hard to understand? |
| What do you think of the layout of the program? | - What do you think about the chapter layout? The pictures? The videos? |
| What do you think of the usability of the program? | - Which parts were easy or hard to use? For example, the navigation, external weblinks, and hand-outs. |
| What do you think of the videos within the online program? | - Was the information provided in the videos clear and understandable? - Are the videos of added value to the program? |
| Do you think you would use the program again? | - If yes, any chapters in particular? - If no, why not? |
| Would you recommend the program to others? | - If yes, to whom? Why? |
| Do you have any points for improvement for the online program? | - Did you miss information? |
