# Supplementary material for: A cross-sectional evaluation of the Dutch RHAPSODY program: online information and support for caregivers of persons with young-onset dementia
Source: Internet Interv. 2022 Mar 26;28:100530. doi: 10.1016/j.invent.2022.100530 (PMC9005959; doi:10.1016/j.invent.2022.100530)
Supplement: Appendix C — User behavior between October and December 2020. [file mmc3.docx]

**Appendix C.**

**Table C.1**: User behavior between October and December 2020

|  | Page views (n) | Unique page views (n) | Average time on page (sec) |
| --- | --- | --- | --- |
| Landing page | 3,027 | 2,461 | 129.8 |
| Main page chapter 1 | 1,256 | 750 | 50.4 |
| YOD explanation | 439 | 360 | 137.5 |
| Medical background | 400 | 344 | 150.7 |
| Diagnostic process | 278 | 242 | 392.8 |
| Heredity of YOD | 190 | 165 | 186.1 |
| YOD-subtypes | 221 | 179 | 129.3 |
| Treatment | 120 | 109 | 204.8 |
| Main page chapter 2 | 428 | 268 | 58.8 |
| Cognitive problems | 285 | 146 | 66.9 |
| Daily activities | 164 | 146 | 265.0 |
| Physical health | 92 | 51 | 34.6 |
| Main page chapter 3 | 402 | 250 | 38.7 |
| Behavioral changes | 138 | 125 | 196.7 |
| Mood changes | 98 | 86 | 126.9 |
| Social behavior | 98 | 81 | 60.6 |
| Lack of initiative | 93 | 73 | 47.6 |
| Physical behavior | 61 | 54 | 88.1 |
| Perceptions on reality | 68 | 59 | 70.6 |
| Main page chapter 4 | 196 | 121 | 36.9 |
| Changes in relationships | 53 | 45 | 126.9 |
| Children | 49 | 47 | 98.6 |
| Communication | 37 | 35 | 56.5 |
| Emotions | 57 | 51 | 62.4 |
| Main page chapter 5 | 127 | 90 | 46.0 |
| Burden | 45 | 41 | 121.5 |
| Balancing caregiving | 45 | 44 | 169.9 |
| Unrealistic expectations | 48 | 43 | 45.3 |
| Activities | 39 | 37 | 51.3 |
| Finding help | 30 | 27 | 63.8 |
| Main page chapter 6 | 133 | 103 | 92.1 |
| YOD healthcare | 28 | 26 | 169.3 |
| Healthcare professionals | 50 | 43 | 82.4 |
| Support for persons with dementia | 41 | 24 | 75.7 |
| Support for caregivers | 40 | 25 | 54.4 |
| Laws | 57 | 50 | 216.9 |
| Regulations | 29 | 26 | 54.3 |
| Nursing home admission | 18 | 17 | 45.8 |
| After passing away | 14 | 14 | 34.6 |
